# 

**Published:** 1815-09

**Authors:** 


### MONTHLY CATALOGUE OF MEDICAL BOOKS.

CHEMICAL Essays, principally relating to the Arts and Manufactures of the British dominions. By Samuel Parks, F.L.S. &c. &c. 12mo.—Baldwin and Co.

Observations on Strictures, and other Affections occasioning a Contraction in the lower Part of the Intestinal Canal; and the Mode of Treatment; accompanied with Cases and Engravings. By W. White. 8vo. Second edition, corrected and enlarged.—Longman and Co.

Elements of Pathology and Therapeutics. By C. H. Parry, M.D. F.R.S. &c. 8vo.—Underwood.

The Vaccine Scourge, Part 2, containing the new Beggar's Opera, alias the Walkerian Farce, alias the London Vaccine Hoax; in Answer to Dr. Walker's Jeneric Opera, a Rod for the Fool's Back. 8vo. Callow.

A Practical and Historical Treatise on Consumptive Diseases, deduced from original Observations, and collected from Authors of all Ages. By Thomas Young, M.D. F.R. and L.S. 8vo.—Underwood.

Facts and Observations on Liver Complaints, and Bilious Disorders in general. By John Faithhorn. 8vo. Second edition, considerably enlarged.—Longman and Co.

---

### *NOTICES TO CORRESPONDENTS.*

*We have frequent complaints from authors that their works are not reviewed. It is not less our wish to show impartiality in selection than in reviewing. But, though the receipt of a copy is by no means a necessary compliment, it may be easily conceived that it must prove a memento. We should not have said so much, were it not that several gentlemen have written to remind us of copies already sent, which have never come to our hands. We are aware how easily such errors may occur in the hurry of publication, but think it right to say thus much in our own vindication.*

*Communications have been received from Drs. Kinglake, St. Clair; Messrs. Bakewell, Battley, Walker, &c. besides several on the Apothecaries' Bill.*

